# Monitoring Physical Behavior in Rehabilitation Using a Machine Learning–Based Algorithm for Thigh-Mounted Accelerometers: Development and Validation Study

**DOI:** 10.2196/38512

**Published:** 2022-07-26

**Authors:** Frederik Skovbjerg, Helene Honoré, Inger Mechlenburg, Matthijs Lipperts, Rikke Gade, Erhard Trillingsgaard Næss-Schmidt

**Affiliations:** 1 Research Unit Hammel Neurorehabilitation Centre & University Research Clinic Hammel Denmark; 2 Department of Clinical Medicine Aarhus University Aarhus Denmark; 3 Department of Medical Information and Communication Technology St. Anna Hospital Geldrop Netherlands; 4 Section of Media Technology Aalborg University Aalborg Denmark

**Keywords:** activity recognition, random forest, acquired brain injury, biometric monitoring, machine learning, physical activity

## Abstract

**Background:**

Physical activity is emerging as an outcome measure. Accelerometers have become an important tool in monitoring physical behavior, and newer analytical approaches of recognition methods increase the degree of details. Many studies have achieved high performance in the classification of physical behaviors through the use of multiple wearable sensors; however, multiple wearables can be impractical and lower compliance.

**Objective:**

The aim of this study was to develop and validate an algorithm for classifying several daily physical behaviors using a single thigh-mounted accelerometer and a supervised machine-learning scheme.

**Methods:**

We collected training data by adding the behavior classes—running, cycling, stair climbing, wheelchair ambulation, and vehicle driving—to an existing algorithm with the classes of sitting, lying, standing, walking, and transitioning. After combining the training data, we used a random forest learning scheme for model development. We validated the algorithm through a simulated free-living procedure using chest-mounted cameras for establishing the ground truth. Furthermore, we adjusted our algorithm and compared the performance with an existing algorithm based on vector thresholds.

**Results:**

We developed an algorithm to classify 11 physical behaviors relevant for rehabilitation. In the simulated free-living validation, the performance of the algorithm decreased to 57% as an average for the 11 classes (F-measure). After merging classes into sedentary behavior, standing, walking, running, and cycling, the result revealed high performance in comparison to both the ground truth and the existing algorithm.

**Conclusions:**

Using a single thigh-mounted accelerometer, we obtained high classification levels within specific behaviors. The behaviors classified with high levels of performance mostly occur in populations with higher levels of functioning. Further development should aim at describing behaviors within populations with lower levels of functioning.

## Introduction

Physical behavior (PB) includes both physical activity (PA) and inactivity, which are both topics of increasing interest in health care. The health benefits associated with PA are well-established [1], which has resulted in the use of PA as prevention and a part of treatment and rehabilitation [2]. The prescription of PA has evolved within a wide range of diseases with long-term health impacts such as diabetes, cardiovascular diseases, obstructive pulmonary diseases, and rheumatoid arthritis [2-6]. Many such subgroups in our societies will continue to need rehabilitation to promote functional recovery, reduce the risk of comorbidities, and prevent the secondary effects of disease [7,8].

In the field of physical and rehabilitation medicine (PRM), functional outcomes and capabilities are of great interest. Today, the International Classification of Functioning, Disability and Health (ICF) is the conceptual foundation of physical and rehabilitation medicine as a biopsychosocial framework for clinicians, researchers, and policy makers [9]**.** Rehabilitation interventions often target functional abilities and limitations to promote physical and cognitive functioning, participation, and the modification of personal and environmental factors [9,10]. These functional aims in daily living require measurement properties that can identify such factors in a meaningful way. Outcome measures used in rehabilitation research are often subjective or self-reported measures [11], which are associated with various limitations such as information bias, intrusiveness, and timeliness [12-14]**,** and more objective measures are warranted. The use of wearable technologies offers an objective and complementary insight to subjective measures. The objective classification and quantification of activities such as standing, sitting, wheelchair ambulation, walking, or running can provide information on changes in functional disability. Additionally, it can indicate changes in more holistic measures, referred to as ICF-related items on activity and participation levels, contextual factors, or transport options such as stair climbing, cycling, and vehicle driving. The development of wearable sensor technologies, such as accelerometers, has added the possibility of monitoring PB continuously for longer periods, making it opportune to investigate the changes and habitual patterns of PB [15,16].

The emerging analytical approaches of raw signal processing use pattern recognition to classify functional activities. Threshold-based algorithms have contributed beneficial frameworks with high accuracies [17]. However, machine-learning techniques have proven useful [18], and many studies have achieved high performance in the classification of physical behaviors through the use of multiple wearable sensors [19-22]. Multiple wearables can be impractical and lead to low compliance [23]; it is necessary to investigate classification potentials that only use 1 sensor device [21,22]. Therefore, the purpose of this study was to further develop and validate a machine learning–based algorithm for thigh-mounted accelerometers. We specifically intended to add the following classes of PB to an existing algorithm: running, cycling, stair climbing, wheelchair ambulation, and vehicle driving.

## Methods

### Design

**Figure 1:**
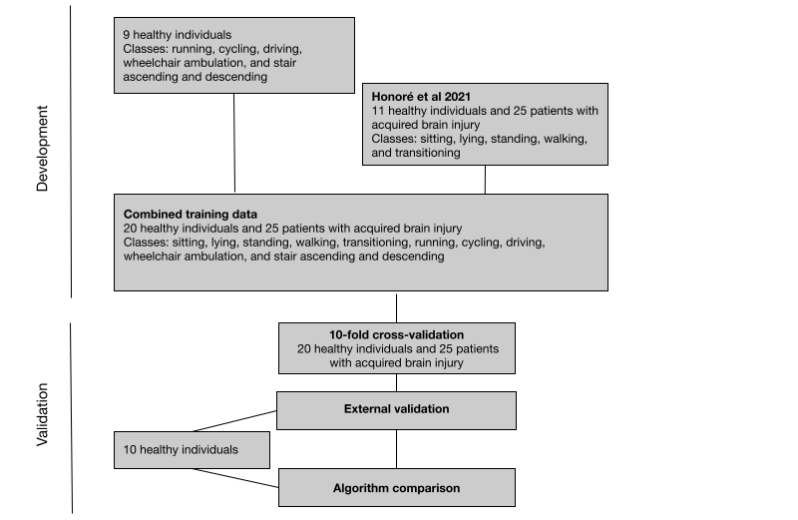
Study overview.

This study was a development and validation study in 2 phases. For a study overview, see Figure 1.

The application of our algorithm was aimed at patients undergoing neurorehabilitation, and the training data collected in the development phase of this study were combined with the training data from a previous study [24], collected in a population of both healthy people and patients with acquired brain injury. The following method section only describes the data collected in this study. The validation phase describes the algorithm developed based on the combined training data from both studies. Due to ethical considerations, the algorithm was validated in a new cohort of healthy individuals, and performance was compared to another algorithm based on vector thresholds [17].

### Instrumentation

A triaxial accelerometer (AX3; Axivity) was mounted on the dominant leg, on the lateral part of the thigh approximately 10 cm above the apex patella. The x-axis was oriented toward the floor in the standing position, as implied by the downward position of the USB port and stated by the visible written information on the device. The accelerometers were programmed with a sampling frequency at 100 Hz, consistent with the method of Honoré et al [24].

### Development Phase

A pragmatic data collection method was applied. A protocol described the positioning, direction, and attachment of the accelerometer. We used 3 taps directly on the accelerometer as a data marker for the start and stop of the recording of behaviors. The participants were asked to perform a minimum of 10 minutes of continuous activity for each PB with the exception of stair climbing. Whenever possible, the behaviors were performed at locations of the participants’ choosing or alternatively, at locations proposed by FS. Instructions were given immediately before each performed behavior, and data were extracted immediately after. Participants contributed the behaviors of convenience and provided information on gender, age, and height (Table 1).

**Table 1:** Description and characteristics of the participants (N=9) contributing training data. The total amount of training data for all participants and the distribution within each activity are reported.

Class		Gender (male, female), n	Age (year), mean (SD)	Height (cm), mean (SD)	Total duration^a^ (h, min)
All participants		4, 5	36.1 (13.4)	176.7 (5.7)	21, 27
Running		4, 2	30.8 (13.0)	179.8 (4.5)	4, 52
Cycling		4, 2	42.8 (14.6)	179.5 (5.2)	6, 10
**Stair climbing**					
	Ascending	3, 2	31.4 (12.6)	178.8 (4.7)	0, 10
	Descending	3, 2	31.4 (12.6)	178.8 (4.7)	0, 9
Driving		2, 2	40 (16.7)	179.2 (5.3)	5, 53
Wheelchair ambulation		3, 2	33 (7.7)	176.8 (5.0)	4, 13

^a^Total duration describes the total amount of training data.

### Data Preprocessing and Learning Scheme

Each activity sequence containing 1 PB was manually identified by the data markers and extracted from the original data file using OMGUI configuration and analysis tool (V43 ; Open Movement). The raw accelerometer data was processed in a custom-made MATLAB script (R2020b; MathWorks) for the manual label annotation of each sample period of 1 second with a sample overlap of 0.5 seconds. All manual annotation and classification were done by FS. For all accelerometer axes, we extracted the features of 1-second samples. Based on the findings of Yan et al [25], a preselected subset of features was used (Textbox 1). To model baseline PB classifications, we used the nonlinear classifier random forest with default hyperparameters in Weka software (version 3.8.4; University of Waikato) [26,27].

**Features used.**

**Features**

- Mean values
- SDs
- Root mean square values
- Maximum number of peaks
- Highest value of axes
- Lowest value of axes
- Number of distinctive points
- Pearson correlation between axes

### Validation Phase

The validation phase consisted of a k-fold cross-validation, an external validation, and an algorithm comparison procedure. To evaluate the potential of the algorithm, we initially performed a stratified 10-fold cross-validation on the training data collected from 9 healthy individuals and the data from Honoré et al [24] from 11 healthy individuals and 25 patients, and the subsets were randomly split. In the external validation, 10 healthy individuals who did not contribute to the training data were asked to participate in the external validation protocol. The protocol consisted of a semistandardized session, where the participants were instructed to carry out a protocol of PBs at a self-determined level of pace, duration, and order, in a setup that enabled the performance of all behaviors. Throughout the session, the participants wore an accelerometer on the thigh and a chest-mounted GoPro camera was used to identify the ground truth of the PBs performed. The video recording was time-synchronized with the accelerometer data using ELAN tool (version 6.4; Max Planck Institute for Psycholinguistics) [28] and was then manually labeled by FS as a criterion measure. Data collected through the external validation protocol were then used as a test set and a second-by-second analysis was conducted by testing the performance of the algorithm in the validation data.

The algorithm for comparison was chosen based on previous use by research institutions in the central regions of Jutland, Denmark [29-33]. We compared the performance of the algorithm by Lipperts et al [17] and our algorithm by analyzing the data collected in the external validation protocol with both algorithms. We reported the results on a total time basis compared to the ground truth and through confusion matrices for both algorithms. In accounting for differences in the available classes between the algorithms, we adjusted our algorithm to only include classes comparable to the classes by Lipperts et al [17]. Therefore, we excluded the implemented wheelchair ambulation and vehicle driving classes, and similarly, we excluded the data parts containing wheelchair ambulation and vehicle driving from the validation sessions. To create a fair basis for comparison, we merged the relevant classes, sitting and lying, to account for sedentary behavior. Additionally, we merged walking, stair climbing, and transitioning under the walking class, corresponding to the walking class by Lipperts et al [17].

### Statistics

For evaluating the performance of the algorithm, we presented confusion matrices for the developed models. We interchangeably used the term performance to refer to the main evaluation metric: F-measure [34,35]. We calculated the F-measure as the harmonic mean between the positive predictive value and sensitivity [36]. In the algorithm comparison, we reported mean errors in durations as calculated by *(|duration^Alg^ – duration^GT^|) / duration^GT^*, where *duration^Alg^* is the total duration of all correctly classified seconds of either algorithm and *duration^GT^* is the duration of the ground truth.

### Ethical Considerations

The study was conducted in accordance with the Helsinki Declaration of 2008 [37], and the General Data Protection Regulation was followed. This study did not require approval from the regional ethics committee, as noninterventional studies do not need approval by the Region Committee on Biomedical Research Ethics in Denmark. We only recruited healthy participants, and written informed consent was obtained from all participants.

## Results

### Participants and Training Data

The data gathering and preprocessing resulted in no missing or exclusion of data. In total, 9 healthy participants contributed data for training the algorithm. Participants of various ages, heights, and gender were included. We strived to accumulate >4 hours of running, cycling, driving, and wheelchair ambulation and 10 sessions of ascending and descending stair climbing (Table 1).

### K-fold Cross-validation

By combining data from Honoré et al [24] with the training data in this study, the algorithm constituted 11 classes of PBs. The initial evaluation by a stratified 10-fold cross-validation (Table 2) showed strong agreement between the labels and the classifications performed by the algorithm, with an average F-measure of 92.8% for all classified PBs—a performance strong enough to be tested in simulated free-living conditions. The performance in classifying running and cycling showed high agreement by reaching F-measures of 100 and 99.6%, respectively. The classification of stair climbing likewise showed promising results by reaching F-measures of 91.4% and 90.2% for ascending and descending stairs, respectively. In discriminating between the 4 behaviors involving similar inactive lower extremity postures, the algorithm showed an F-measure of 92.7% for sitting and 92.3% for lying, whereas driving and wheelchair ambulation reached 99.4% and 98.9%, respectively. Walking and standing yielded F-measures of 89% and 96.3%, respectively. Transitioning resulted in the lowest F-measure of 72.5%.

**Table 2:** Confusion matrix from stratified 10-fold cross-validation. Correctly and incorrectly classified seconds of physical behavior by the algorithm (columns) and the ground truth (rows). Seconds overlap by 0.5 second.

Ground truth	Algorithm										
	Sitting	Transitioning	Walking	Standing	Lying	Ascending stairs	Cycling	Descending stairs	Running	Driving	Wheelchair ambulation
Sitting	2236	59	0	0	68	0	2	0	0	64	106
Transitioning	27	1683	286	46	32	5	104	5	0	92	163
Walking	0	220	3103	21	0	13	15	15	0	0	0
Standing	0	48	4	1688	5	0	3	0	0	0	0
Lying	17	48	0	0	1935	0	0	0	0	64	51
Ascending stairs	0	7	63	0	0	1060	31	24	6	0	0
Cycling	0	36	20	1	0	19	44,280	7	0	1	53
Descending stairs	0	0	105	0	0	30	12	979	7	0	0
Running	0	0	7	0	0	1	1	8	34,985	0	0
Driving	4	44	0	0	20	0	3	0	0	42,148	109
Wheelchair ambulation	5	52	0	0	19	0	28	0	0	80	30,134

### External Validation

The external validation protocol resulted in 10 sessions of PB monitoring, which included all the behaviors of interest performed by 10 healthy participants recruited at Hammel Neurorehabilitation Center and University Research Clinic, Denmark. Participant characteristics are described in Table 3. The performance of the algorithm in the validation data showed moderate agreement between the ground truth and the classifications by the algorithm with 57% as the average F-measure for all classifications (Table 4). The performance in classifying running and cycling remained high by reaching 88.7% and 87.1%, respectively. The classification of stair climbing decreased to an F-measure of 44.8% for ascending and 25.5% for descending stair climbing. In discriminating between the 4 behaviors involving inactive lower extremity postures, the algorithm showed an F-measure of 63.7% for sitting, 66.8% for lying, 77.1% for driving, and 31% for wheelchair ambulation. Walking, standing, and transitioning were classified with F-measures of 55%, 67.1%, and 20%, respectively.

**Table 3:** Characteristics of participants contributing data from the external validation.

Characteristic	Value
Participants, n	10
Gender (male, female), n	5, 5
Age (year), mean	43.6
Height (cm), mean	174.4
Duration^a^ (min, sec), mean	12, 58

^a^Duration describes the average time taken to complete the validation session.

**Table 4:** Confusion matrix from the external validation. Correctly and incorrectly classified seconds of physical behavior by the algorithm (columns) and the ground truth (rows). Seconds overlap by 0.5 second.

Ground truth	Algorithm											
	Sitting	Transitioning	Walking	Standing	Lying	Ascending stairs	Cycling	Descending stairs	Running	Driving	Wheelchair ambulation	
Sitting	746	28	15	0	236	2	10	5	0	163	151	
Transitioning	1	131	64	0	10	4	66	14	7	35	30	
Walking	5	253	1178	72	0	60	108	191	89	50	69	
Standing	1	190	118	589	1	31	47	23	16	8	3	
Lying	208	58	4	0	746	0	0	0	0	54	136	
Ascending stairs	0	8	143	29	0	184	40	38	42	0	0	
Cycling	0	57	57	6	0	19	1673	13	12	5	18	
Descending stairs	0	17	520	26	0	30	7	162	12	0	0	
Running	0	13	28	7	0	4	5	18	1014	0	0	
Driving	23	50	31	0	34	0	4	15	4	3124	542	
Wheelchair ambulation	1	140	52	0	0	4	23	16	2	830	453	

### Algorithm Comparison

To compare the performance of the 2 algorithms, noncomparable classes were excluded. The validation sessions subsequently averaged 7.21 minutes and included the behaviors lying, sitting, standing, transitioning, walking, stair climbing, running, and cycling. The results of the merged algorithm showed high performance by reaching an averaging F-measure of 85.3% for all classes in the external validation data (Table 5). In comparison, Lipperts et al’s [17] algorithm showed an average F-measure of 81.1% (Table 6). Table 7 shows the mean error by the algorithms for each behavior class across the 10 validation sessions. The results indicated high agreement between the ground truth and both algorithms when classifying sedentary behavior, walking, running, and cycling, whereas both algorithms showed poor performance in classifying standing. The mean error for Lipperts et al’s [17] algorithm varied between 13.6% to 72.8%, consequently overestimating sedentary and standing behavior, and was hardly influenced by not detecting running and cycling in 2 and 1 sessions of validation, respectively. The mean error for our algorithm varied between 7.9% to 41.7%, consequently underestimating all classes.

**Table 5:** Confusion matrix from the adjusted algorithm in external validation data. Correctly and incorrectly classified seconds of physical behavior by the algorithm (columns) and the ground truth (rows). Seconds overlap by 0.5 second.

Ground truth	Algorithm				
	Sedentary	Walking	Standing	Cycling	Running
Sedentary	2046	143	0	11	0
Walking	10	2381	95	122	95
Standing	0	359	568	40	16
Cycling	0	191	6	1631	8
Running	0	66	7	6	1010

**Table 6:** Confusion matrix for Lipperts et al’s [17] algorithm in the external validation data. Correctly and incorrectly classified seconds of physical behavior by the algorithm (columns) and the ground truth (rows). Seconds overlap by 0.5 second.

Ground truth	Algorithm				
	Sedentary	Walking	Standing	Cycling	Running
Sedentary	2124	4	72	0	0
Walking	219	1999	443	12	30
Standing	28	156	776	12	11
Cycling	0	122	205	1491	7
Running	0	203	43	0	834

**Table 7:** Mean error, SD, and range of output duration parameters for analyzing the external validation data by the 2 algorithms. We calculated the mean error, SD, and minimum and maximum error percentage across the 10 validation sessions within each activity class.

Algorithm, parameter		Activities				
		Sedentary	Standing	Walking	Running	Cycling
**Lipperts et al [17]**						
	Mean error (%)	13.6	72.8	14.5	27.2	21.8
	SD (%)	7.2	72.8	6.2	40.9	29.8
	Minimum error (%)	6.4	22.2	2.9	1.6	1.3
	Maximum error (%)	28.6	267	22.2	100	100
**Skovbjerg et al**						
	Mean error (%)	7.9	41.7	12.4	10	8.1
	SD (%)	4	14.1	7	15.6	5.3
	Minimum error (%)	2.4	19	2.8	0	0
	Maximum error (%)	13.9	59.1	23	51.5	16.6

## Discussion

### Principal Findings

We developed an algorithm to classify 11 PBs related to daily living in rehabilitation. The cross-validation demonstrated high performance (93%), and the validation of the algorithm in a free-living setting was reasonable. The algorithm showed moderate performance (57%) when applied to simulated free-living data. The algorithm performed well in classifying cycling and running, whereas an acceptable level of performance was found in classifying driving. In classifying the remaining behaviors, the algorithm showed low to moderate performance ranging from 20% to 67%. In comparison to a validated algorithm by Lipperts et al [17], our adjusted algorithm showed equally strong performance and high agreement with ground truth annotations after merging relevant classes. The significant performance decrease between cross-validation and external validation may be explained by the fact that in the cross-validation, different samples from the same individual were included in both training and test splits. In the external validation, the individuals and their specific motion pattern were not included in the training data.

### Discriminating Rehabilitation Relevant Physical Behaviors

The behaviors classifiable by the algorithm were based on the rationale and aims of rehabilitation. Our results showed lower performance in discriminating behaviors performed in sitting postures, which can be explained by their similar body positioning and behavioral characteristics. Although discriminating these behaviors is important when considering activity and participation from an ICF perspective, the differences within sitting, wheelchair ambulation, and driving might be clinically irrelevant from a perspective of monitoring PA and energy expenditure at a body function and anatomy level. In a visual inspection of accelerometer data, signals from the 3 behaviors revealed only insignificant differences. Likewise, the algorithm had difficulties discriminating between the PBs by the accessible features. Overall, the algorithm performed better in discriminating behaviors with larger variations in body position and movement trajectories, mostly occurring in patients with higher levels of functioning.

### Comparison to Existing Literature

Pavey et al [38] achieved a 93% overall accuracy for classifying the PBs—sedentary, stationary, walking, and running—using a wrist-worn accelerometer with the random forest classifier in laboratory settings among 21 healthy participants, evaluated using leave-one-subject-out cross-validation. A back validation in free-living using activPAL as a reference standard for stepping versus nonstepping showed high agreement. Alber et al [39] used a waist-worn accelerometer for classifying lying, standing, sitting, walking, wheelchair ambulation, and stair climbing among 13 subjects with incomplete spinal cord injury, using a support vector machine (SVM) classifier. Their laboratory-based algorithm decreased from 92% to 55% when tested on home-based data, whereas their home-based algorithm reached 86%, evaluated using within-subject cross-validation.

When focusing on single thigh-mounted accelerometry, Awais et al [20] reached a mean F-measure ranging from 68% to 76% with different combinations of features, using SVM classifier in identifying sitting, lying, standing, and walking among 20 older people in free-living conditions evaluated using leave-one-subject-out cross-validation. Likewise, Tang et al [22] investigated the number of sensors and found a mean F-measure of 76% using a single thigh-worn accelerometer and SVM classifier in identifying sitting, lying, and standing among 42 healthy participants in semistandardized laboratory settings, evaluated using leave-one-subject-out cross-validation. In comparison to Tang et al [22] and Awais et al [20], we reached an F-measure of 57%, evaluated using simulated free-living conditions with 11 classes of PB. For the abovementioned studies, they all use fewer classes of activities, which expectedly will increase the performance of an algorithm and might explain why our algorithm does not reach their level. As indicated in the algorithm comparison, the level of performance required for valid estimation can be obtained by merging relevant classes. It will compromise the degree of details but simultaneously add the possibility of adjusting the measures of PB in relation to the aims.

### Algorithm for Patients With Acquired Brain Injury

Our algorithm was aimed at patients undergoing neurorehabilitation. Classifying behaviors within subgroups potentially exposed to characteristic movement patterns, the behavior classes—sitting, lying, standing, walking, and transitioning—were partly based on training data from the population of interest [24]. Some specific PBs or movement patterns such as transitioning and walking may be more influenced by disease-specific characteristics than others. Similarly, some PBs can be less prone to disease-specific characteristics depending on functional level or disease severity. Using healthy individuals for training the algorithm relies on the rationale that a higher functional level is required to perform PB, such as running, and hence is associated with a movement pattern comparable to movement patterns in healthy individuals. Adversely, PBs, such as wheelchair ambulation, may be independent of specific movement characteristics. In principle, the training data should be gathered in the target population to capture complex movements influenced by disabilities, although it can be argued that activities less prone to disease-specific characteristics can be gathered in healthy populations due to ethical considerations.

### Limitations

The training data for this study was collected in a setup similar to a laboratory setting. Although the PBs were performed in a free-living setting, only 1 PB was recorded in each session, and therefore, the composition of PBs in free-living was not reflected in the training data. Our training data were probably influenced by a severe class imbalance between the newly gathered classes and the classes gathered in Honoré et al [24], which might have affected the performance of the algorithm in the validation data. Less available training data decrease the performance by reducing the ability of a classifier to generalize patterns not seen before. Balancing minority classes through supplementary data gathering might be advantageous in future work. We did not include a free-living validation but designed a semistandardized session aimed at simulating free-living. All validation sessions were conducted in the same environment—they only lasted 10-20 minutes, and the participants were enforced to perform PBs corresponding to the classes of the algorithm. Variation between sessions consisted of the order and duration of the behaviors. We used video recordings as a criterion measure for labeling accelerometer signals and further merged annotation definitions with Honoré et al [24] to align the labeling protocol, thus the ground truth labeling was only performed by FS and the reliability was not evaluated. The algorithm comparison procedure might have been influenced by differences in annotation definitions, leading to an underestimation of the performance by Lipperts et al’s [17] algorithm. Likewise, the cropping procedure have introduced minor differences in the data analyzed by each algorithm.

### Clinical Implications

The algorithm comparison revealed that our merged algorithm, constituting 5 classes, reached an acceptable level of agreement with both the algorithm of Lipperts et al [17] and the ground truth. However, the 11-class algorithm did not show acceptable levels of performance within all classes, indicating that the number of behavior classes and similarities between classes may influence the obtainable level of performance. To monitor physical behavior within various functional levels of patients undergoing neurorehabilitation, further research and changes in the monitor setup are required to attain the desired levels, especially within wheelchair ambulation. Furthermore, this study provided an external validation performed in a simulated free-living setting, which constitutes an estimate of the algorithm’s performance in clinical settings.

### Conclusion

We developed an algorithm for classifying rehabilitation-relevant physical behaviors. We successfully added the classes of running and cycling, which were classified with high performance in a simulated free-living setting. Furthermore, we added stair climbing, wheelchair ambulation, and vehicle driving, which showed high performance in the 10-fold cross-validation on training data, but low to moderate performance in the free-living setting for new individuals. Increasing the implications for rehabilitation use might be done by focusing on the performance in classifying behaviors within populations with lower levels of functioning and within transport ambulation and the use of assistive devices.

## Abbreviations

ICF: International Classification of Functioning, Disability and Health
PA: physical activity
PB: physical behavior
SVM: support vector machine
